# Supplementary material for: Conditional chemoconnectomics (cCCTomics) as a strategy for efficient and conditional targeting of chemical transmission
Source: eLife. 2024 Apr 30;12:RP91927. doi: 10.7554/eLife.91927 (PMC11060718; doi:10.7554/eLife.91927)
Supplement: Figure 3—source data 3. [file elife-91927-fig3-data3.zip › Figure 3 source data3 part 1.zip!/Figure 3-Source data 3-part1/1082-9-CRISPResso_Report_zod2ewbs.zip!/CRISPResso_on_1082_11/9.Alleles_frequency_table_around_sgRNA_catccaggcagatccaggtg.pdf]

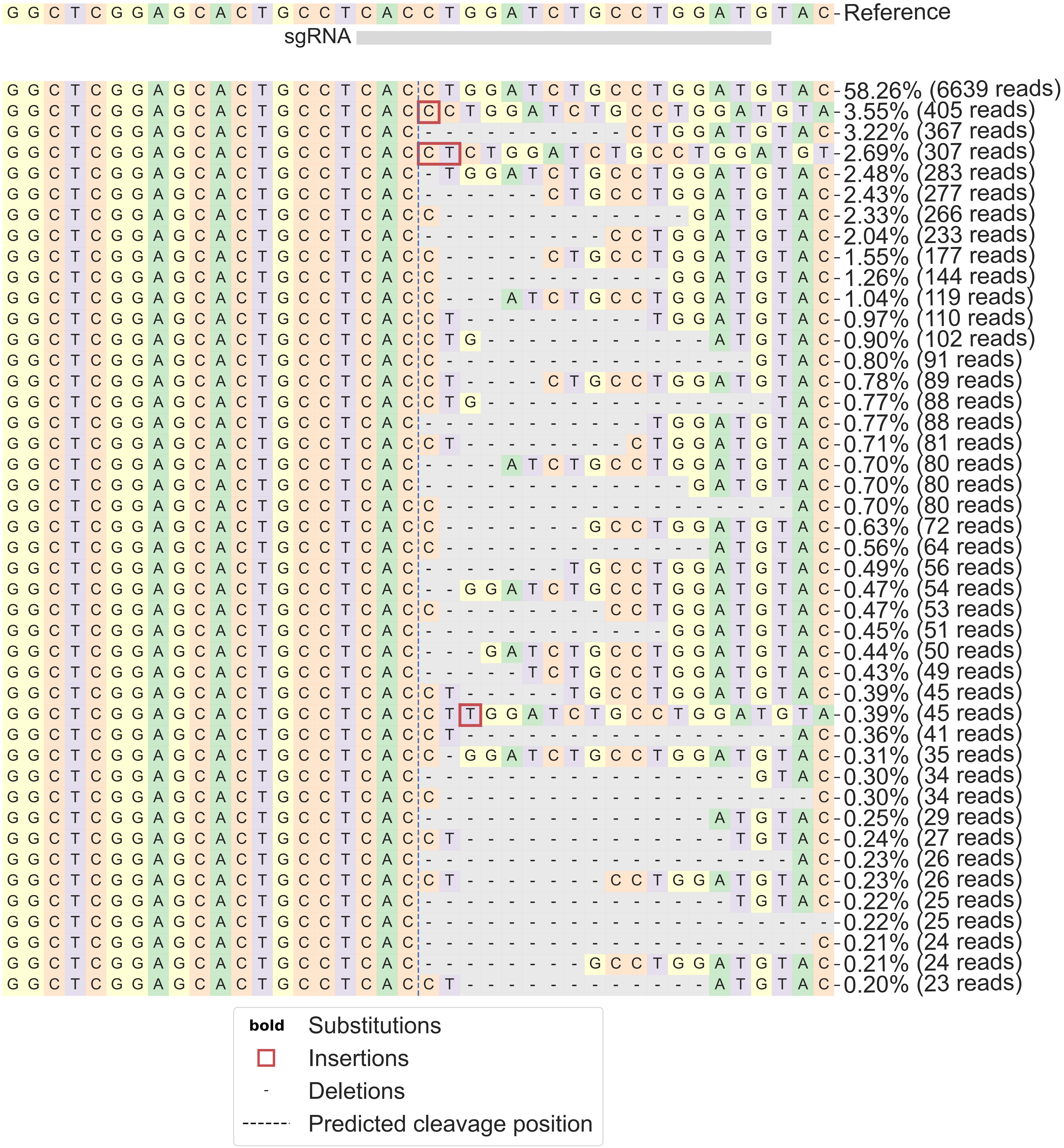
G G C T C G G A G C A C T G C C T C A C C T G G A T C T G C C T G G A T G T A C Reference
sgRNA
58.26% (6639 reads)
3.55% (405 reads)
3.22% (367 reads)
2.69% (307 reads)
2.48% (283 reads)
2.43% (277 reads)
2.33% (266 reads)
2.04% (233 reads)
1.55% (177 reads)
1.26% (144 reads)
1.04% (119 reads)
0.97% (110 reads)
0.90% (102 reads)
0.80% (91 reads)
0.78% (89 reads)
0.77% (88 reads)
0.77% (88 reads)
0.71% (81 reads)
0.70% (80 reads)
0.70% (80 reads)
0.70% (80 reads)
0.63% (72 reads)
0.56% (64 reads)
0.49% (56 reads)
0.47% (54 reads)
0.47% (53 reads)
0.45% (51 reads)
0.44% (50 reads)
0.43% (49 reads)
0.39% (45 reads)
0.39% (45 reads)
0.36% (41 reads)
0.31% (35 reads)
0.30% (34 reads)
0.30% (34 reads)
0.25% (29 reads)
0.24% (27 reads)
0.23% (26 reads)
0.23% (26 reads)
0.22% (25 reads)
0.22% (25 reads)
0.21% (24 reads)
0.21% (24 reads)
0.20% (23 reads)
bold Substitutions
Insertions
Deletions
Predicted cleavage position
